# Could an Increased Percentage of Immature Granulocytes Accompanying Dyspepsia Predict COVID-19?

**DOI:** 10.3390/medicina58101460

**Published:** 2022-10-15

**Authors:** Kadir Gisi, Sukru Gungor, Murat Ispiroglu, Bulent Kantarceken

**Affiliations:** 1 Department of Gastroenterology, Sutcu Imam University, Kahramanmaras 46050, Turkey; 2 Department of Pediatric Gastroenterology, Sutcu Imam University, Kahramanmaras 46050, Turkey

**Keywords:** COVID-19, digestive symptom, dyspepsia, immature granulocyte

## Abstract

*Background and Aim*: Although vaccination practices continue at a fast pace around the world, the severe acute respiratory syndrome coronavirus 2 (SARS-CoV-2) still threatens people’s lives. In this study, we aimed to determine the presence of SARS-CoV-2 in patients who do not have the typical symptoms of the novel coronavirus disease 2019 (COVID-19), but only present with dyspepsia, and to investigate the role of immature granulocytes in the early diagnosis of these patients. *Material and Methods*: Adult and pediatric patients suffering from dyspepsia were included in the study. The patients were divided into two groups, ‘‘positive’’ and ‘‘negative’’, based on their SARS-CoV-2 polymerase chain reaction test results. Immature granulocyte count (IG), immature granulocyte percentage (IG%), C-reactive protein (CRP), and neutrophil-to-lymphocyte ratio (NLR) values were recorded. *Results:* A total of 238 patients, including 25 (10.5%) pediatric and 213 (89.5%) adult patients, were included in the study. A total of 2 (8%) pediatric patients and 17 (7.9%) adult patients tested positive for SARS-CoV-2. The IG, IG%, and CRP parameters were significantly higher in the SARS-CoV-2-positive patients compared to the SARS-CoV-2-negative patients. The optimal cut-off value predictive of COVID-19 infection was determined to be ≥0.650 (sensitivity: 52.6% and specificity: 95.5%, *p* = 0.001) for IG%. *Conclusions:* It should be noted that dyspepsia may also be a COVID-19 symptom. IG% values, which can be determined with a hemogram test, a cheap and easily accessible test, may be a warning in the early detection of patients who do not have the typical symptoms of COVID-19.

## 1. Introduction

Coronavirus pneumonia cases caused by the severe acute respiratory syndrome coronavirus 2 (SARS-CoV-2) broke out in the city of Wuhan in the Hubei province of China in early December 2019 and then spread rapidly all around the world [1]. The first novel coronavirus disease 2019 (COVID-19) case in Turkey was documented on 11 March 2020, and the pandemic was declared on the same day by the World Health Organization [2]. As occurred all around the world, immediately after the first case was declared in Turkey, changes and restrictions began to be implemented in daily life as the spread of the virus increased. COVID-19 acts similar to a systemic disease with a variable range of severity, from the asymptomatic clinical form to respiratory failure and increased mortality. COVID-19 is characterized by interstitial pneumonia and vascular damage that may lead to severe adverse outcomes, and sometimes it can be fatal [3,4]. Studies have shown that 1/10 of COVID-19 patients may only have gastrointestinal symptoms without any respiratory symptoms, which will delay their diagnosis [5]. Among the gastrointestinal symptoms identified in adult COVID-19 patients, diarrhea is the most common, followed by nausea/vomiting, and abdominal pain, and vomiting is reported to be the most common symptom in pediatric patients [6].

Moreover, restrictions upon daily life have led to stress and anxiety in people. Since dyspepsia is a stress-sensitive disorder, stress and anxiety are known to trigger and exacerbate dyspepsia [7]. A complete blood count (hemogram) is one of the commonly requested tests for patients admitted to adult and pediatric gastroenterology outpatient clinics with the complaint of increased dyspepsia [8]. Immature granulocytes (IG), which can be measured in complete blood counts, are the common name used for granulocyte (neutrophil) precursors, namely myelocytes, promyelocytes, and metamyelocytes, which are not found in peripheral blood except for during the neonatal period. IG counts and immature granulocyte percentages (IG%) can easily be obtained by performing a routine complete blood count test, thanks to technological advancements in automated hemogram devices [7]. The detection of immature granulocytes in peripheral blood is an indicator of bone marrow activation and severe infection. In recent studies, immature granulocytes have been shown to be an effective marker for predicting infection severity [9,10]. Finally, given the dynamic characteristic of the COVID-19 pandemic, the relationship between the prognosis of SARS-CoV-2 infection and digestive symptoms is still a matter of debate [11]. Additionally, in those patients with digestive symptoms, there are currently no early diagnostic markers to alert the clinician in simple laboratory tests unless the clinician suspects SARS-CoV-2 infection.

In this study, we aimed to determine the presence of SARS-CoV-2 infection in patients who did not have the typical symptoms of COVID-19 but only presented with dyspepsia and investigate the role of immature granulocytes in the early diagnosis of such patients.

## 2. Material and Methods

Patients presenting to pediatric and adult gastroenterology outpatient clinics with the complaint of persistent dyspepsia between August 2020 and December 2020 were included in the study. The demographic and laboratory data of the included patients were retrospectively screened and recorded.

All procedures performed in the studies involving human participants were performed in accordance with the ethical standards of the institutional and/or national research committee and the 1964 Declaration of Helsinki and its later amendments or comparable ethical standards. Approval was obtained from the local ethics committee of Sutcu Imam University before starting the study, with a decision date of 25 January 2021, session 2021/04, no. 10.

### 2.1. Inclusion Criteria

Pediatric and adult patients who had dyspeptic complaints resistant to diet and medical treatment, who had no complaints other than dyspepsia, who were scheduled for endoscopy, and who underwent COVİD-19 PCR tests were included in the study.

### 2.2. Exclusion Criteria

Patients who underwent endoscopy with indications other than resistant dyspeptic complaints, such as variceal bleeding, foreign body ingestion, and corrosive substance ingestion, and patients who had not taken a PCR test for COVID-19 were excluded from the study.

### 2.3. Laboratory Data Evaluated in the Study

The results of SARS-CoV-2 Polymerase Chain Reaction (PCR) tests, complete blood counts, white blood cell counts (WBCs), neutrophils, lymphocytes, hemoglobin levels, immature granulocyte counts, immature granulocyte percentages, and C-reactive protein (CRP) tests, which were routinely checked before endoscopy, were recorded.

### 2.4. Groups

The patients were divided into two groups (SARS-CoV-2 positive vs. SARS-CoV-2 negative), based on their PCR test results. Demographic and laboratory data were compared for these two groups.

### 2.5. Complete Blood Count Data Analysis

The complete blood count results were studied using an automated hematological analyzer (XN 3000; Sysmex Corp., Kobe, Japan), and WBC counts, neutrophil counts, lymphocyte counts, IG counts, and IG% values were measured. Neutrophil-to-lymphocyte ratios (NLR) were calculated manually. IG% was calculated as the ratio of the IG count value to the white blood cell count value.

### 2.6. SARS-CoV-2 PCR Analysis

Nasal and pharyngeal swab samples collected from the patients were examined with the CFX96 Touch Real-Time Polymerase Chain Reaction (RT-PCR) detection system using the Bio-speedy (Bioeksen) RT-PCR kit provided by the Turkish Ministry of Health.

### 2.7. Statistical Analysis

The statistical analyses were performed using statistical software (SPSS 22 for Windows, Chicago). Descriptive statistics are expressed as mean ± standard deviation for the continuous variables and as percentages (%) for the categorical variables. The chi-squared test and the Fisher’s exact test were conducted for the categorical variables. The Shapiro–Wilk test was performed to check the normality of the distribution of the data for the continuous variables. The independent samples *t*-test was used to determine the mean differences of a dependent variable between two independent groups and whether there was a significant difference in the normally distributed parameters. The Mann–Whitney *U* test was used to compare the nonnormally distributed continuous variables. Correlation analysis was conducted to show the direction and strength of the relationship between two numeric variables. If the data were distributed normally, the Pearson correlation coefficient was preferred, whereas the Spearman’s Rank correlation coefficient was preferred for the nonnormally distributed data. The numeric data are given as median ± standard deviation (minimum–maximum values), and the categorical data are shown as frequencies (n) and percentages (%). In the analyses, *p* < 0.05 was considered statistically significant.

We performed an ROC curve analysis to determine the best cut-off values of the hematological parameters that could predict the development of COVID-19 infection. We conducted a risk analysis with logistic regression according to the cut-off values that we found. Results with a p-value of <0.05 were considered statistically significant.

## 3. Results

A total of 238 patients, including 25 (10.5%) pediatric and 213 (89.5%) adult patients, were included in the study. The mean age of the patients was 41.08 ± 19.16 years. The mean age of the pediatric patients was 9.35 ± 5.37, and the mean age of the adult patients was 44.81 ± 16.57. A total of 11 (44%) of the pediatric patients were female, and 14 (56%) were male, while 95 (44.6%) of the adult patients were female, and 118 (55.4%) were male. A total of 2 (8%) pediatric patients and 17 (7.9%) adult patients were determined to be positive for SARS-CoV-2. There was no significant difference in terms of sex or age distributions between the patients with and without SARS-CoV-2 (*p* = 0.482, *p* = 0.269, respectively). A total of 100 (45.7%) of the SARS-CoV-2-negative patients were female, and 119 (54.3%) were male. A total of 6 (31.6%) of the SARS-CoV-2-positive patients were female, and 13 (68.4%) were male. There was no significant difference in terms of sex or age between the patients with and without SARS-CoV-2 infection (*p* = 0.236, *p* = 0.269, respectively) (Table 1).

There was no significant difference between the SARS-CoV-2-positive and the SARS-CoV-2-negative groups in terms of their WBC, Hb, neutrophil, lymphocyte or NLR values (*p* = 0.637, *p* = 0.478, *p* = 0.513, *p* = 0.739, and *p* = 0.752, respectively). The IG, IG%, and CRP parameter values were statistically significantly higher in the SARS-CoV-2-positive patients in comparison to the SARS-CoV-2-negative patients (*p* = 0.008, *p* = 0.042, and *p* = 0.043, respectively) (Table 1).

There was no statistically significant difference in terms of WBC or NLR values between the SARS-CoV-2-positive and -negative pediatric patients (*p* = 0.847 and *p* = 0.856, respectively). The CRP, IG, and IG% values were statistically significantly higher in the SARS-CoV-2-positive pediatric patients compared to the SARS-CoV-2-negative pediatric patients (*p* < 0.001, *p* = 0.046, and *p* < 0.001, respectively). Additionally, there was no statistically significant difference in terms of WBC or NLR values between the SARS-CoV-2-positive and -negative adult patients (*p* = 0.849 and *p* = 0.850, respectively). However, the CRP, IG, and IG% values were statistically significantly higher in the SARS-CoV-2-positive adult patients compared to the SARS-CoV-2-negative adult patients (*p* = 0.008, *p* = 0.014, and *p* = 0.009, respectively) (Table 2). The correlations between the CRP, IG, and IG% values, which were higher in the SARS-CoV-2-positive patient group, were evaluated with the Pearson correlation test. Accordingly, there was a weakly positive significant correlation between CRP and GI values (r: 0.279, *p* = 0.014), and there was a statistically significant positive correlation between CRP and IG% values (r: 0.892 *p* < 0.001) (Table 3).

The best cut-off points of the hematological parameters for the diagnosis of SARS-CoV-2 infection in the patients presenting with the complaint of dyspepsia were determined by an ROC curve analysis. The optimal cut-off point predictive of SARS-CoV-2 infection was determined to be ≥0.045 (sensitivity: 68.4% and specificity: 85.4%, *p* = 0.001) for IG, ≥0.650 (sensitivity: 52.6% and specificity: 95.5%, *p* = 0.001) for IG% and ≥12.4 (sensitivity: 63.2% and specificity: 91.4%, *p* = 0.020) for CRP (Table 4). After we performed a risk analysis using a logistic regression model based on these cut-off points for COVID-19 diagnosis only in the patients presenting with the complaint of dyspepsia, we demonstrated that the risk increased 8.546 times (*p* < 0.001) in the patients with IG values of ≥0.045 × 10^9^ L, 23.611 times (*p* < 0.001) in the patients with IG% values of ≥0.650, and 18.171 times (*p* < 0.001) in the patients with CRP values of ≥12.4 mg/L (Table 5).

## 4. Discussion

Our study showed that COVID-19 infection may present only with resistant dyspeptic complaints in every age group. Additionally, it showed that CRP, IG, and IG% values were higher in the SARS-CoV-2-infected patient group compared to the noninfected group. These findings may guide clinicians in the early diagnosis of COVID-19 cases. SARS-CoV-2 infections have quickly spread all around the world shortly since the disease emerged in China, and a pandemic was declared by the WHO in March 2020 [2]. The main symptoms of COVID-19 were known to be coughing, fever, and shortness of breath in the beginning, but as the spread of the virus increased, the variety of symptoms began to increase, and patients have been diagnosed with COVID-19 with unexpected complaints [12]. Recent studies revealed that SARS-CoV-2 may also be spread via feces [13]. However, it was also demonstrated that SARS-CoV-2 infection may also emerge with gastrointestinal symptoms after it was determined that SARS-CoV-2 had the ability to bind to ACE2 receptors in the gastrointestinal tract [14]. The incidence of gastrointestinal symptoms in COVID-19 patients ranges from 3% to 79% [15]. Diarrhea, nausea/vomiting and weight loss are the most commonly seen gastroenterological symptoms in adult patients, while vomiting is the most common symptom reported in children. In a study of 46 COVID-19 cases with no symptoms or mild symptoms, the frequency of gastrointestinal symptoms was found to be 35%, and the frequency of dyspepsia was reported as 11% [16]. Therefore, it should be highlighted that extra care should be exercised in terms of COVID-19 when it comes to patients presenting with complaints other than respiratory symptoms [6,17].

To the best of our knowledge, our study is the only study emphasizing concurrent SARS-CoV-2 in patients presenting with the complaint of persistent dyspepsia. In our study, 19 (8%) of the 238 patients who underwent endoscopy due to persistent dyspepsia were determined to be positive for SARS-CoV-2. None of the patients who were determined to have SARS-CoV-2 had any complaints other than dyspepsia. The course of SARS-CoV-2 infection is known to be much more severe in patients with comorbidities and in geriatric patients. Although there are studies reporting that the prevalence of infection is higher in the male sex, there are also studies reporting that there is no significant correlation between SARS-CoV-2 infection and sex [18,19]. In our study, there was no statistically significant difference in terms of sex or age between the patients with and without SARS-CoV-2 infection. It was shown in a survey study, which was conducted in Japan and which included adult patients, that the COVID-19 pandemic made existing symptoms worse in 20% of patients with functional dyspepsia. It was stated in the same study that stress caused by SARS-CoV-2 was effective in worsening the existing symptoms of the patients [20]. Additionally, in a study in Italy, it was stated that functional gastrointestinal symptoms increased, especially in children and young people during the pandemic period [21]. We think that both the SARS-CoV-2 infection itself and the stress caused by COVID-19 may lead to dyspepsia or aggravate existing dyspepsia in the patients on whom we performed endoscopies. IG, IG%, and NLR values were demonstrated to increase in infectious and noninfectious inflammatory diseases, such as malignancies [22,23,24,25]. Studies have also suggested that IG% is a more predictive marker in the early diagnosis of any infection as compared to procalcitonin [26] and CRP [27] values, which are well-known markers that have been previously studied. It has been emphasized in various studies that IG% may be used in the early diagnosis of acute appendicitis, acute cholecystitis, acute pancreatitis, sepsis, and neonatal sepsis in pediatric patients [22,28,29,30,31]. Additionally, Ayres et al. [30] stated that sepsis could be ruled out in cases where the IG% value is detected as being lower than 2. In another study, Unal et al. [31] reported that IG% was an effective and inexpensive method for use in the early detection of acute necrotizing pancreatitis. None of the patients included in our study had a systemic inflammatory response (SIRS) or sepsis. In contrast to the aforementioned reports, we found that the IG and IG% levels were significantly higher in the patients with SARS-CoV-2 infection as compared to the SARS-CoV-2-negative patients.

Similarly, it was emphasized in some studies that NLR can be a useful prognostic biomarker for the detection of inflammatory diseases and sepsis [32]. Nalbant et al. [33] stated that NLR may be an independent predictive factor for the diagnosis of COVID-19 infection. However, in our study, there was no significant difference in terms of the NLR values between the SARS-CoV-2-positive and SARS-CoV-2-negative patients. This result may have occurred due to the absence of clinical symptoms and signs suggesting sepsis and/or inflammatory disease other than dyspepsia or early-stage COVID-19 in the patients we included in our study. Van der Geest et al. [34] reported that IG% and CRP may be interchangeable, while combining CRP and IG% findings provides better results for predicting microbial infection. In our study, the CRP values were determined to be significantly higher in the SARS-CoV-2-positive patients in comparison to the SARS-CoV-2-negative patients. There were significant positive correlations between the CRP values and the IG and IG% values (r: 0.279, p: 0.014; r: 0.892 *p* < 0.001, respectively).

Ha et al. [35] determined an IG% cut-off point of 0.5 for the differentiation of complicated and uncomplicated sepsis. In another study, the cut-off point for IG% was determined to be 0.4 (sensitivity: 69.8% and specificity: 29.5%) in predicting sepsis based on clinical diagnosis [28]. There is no diagnostic study in the literature which was conducted with IG and IG% to predict SARS-CoV-2 infection in patients presenting only with the complaint of dyspepsia. Therefore, we determined the optimal cut-off points for CRP, IG, and IG% with an ROC curve analysis to predict SARS-CoV-2 infection in our study. Additionally, in contrast with the literature, we performed a risk analysis for SARS-CoV-2 infection using logistic regression analysis according to these cut-off points.

### Limitations

Our study included both pediatric and adult patients. Another strength of our study is that it is the first study to develop an early diagnostic marker of COVID-19 infection in patients with dyspepsia using frequently used hematological data. The retrospective nature of the study, the low number of pediatric patients, and the low number of patients with COVID-19 infection were the limitations of our study.

## 5. Conclusions

It should be noted that dyspepsia may also be a symptom in SARS-CoV-2 infected patients. In this period when the world cannot completely rid itself of the COVID-19 pandemic, our study showed that dyspepsia, a nonspecific complaint for COVID-19, can be suspected and diagnosed early with simple hematological data, such as immature granulocyte percentages and counts. However, these findings need to be supported by larger and more comprehensive studies.

## Figures and Tables

**Table 1:** Comparison of laboratory data of patients with and without SARS-CoV-2 infection.

	SARS-CoV-2 Negative All Patients (219)	SARS-CoV-2 Positive All Patients (19)	
	n (%)	n (%)	*p* *
Gender			0.236
Female	100 (45.7)	6 (31.6)	
Male	119 (54.3)	13 (68.4)	
	Mean ± SD	Mean ± SD	*p* **
Age	41.45 ± 19.15	36.41 ± 19.15	0.269
WBC (10^9^ L)	7.80 ± 2.83	7.42 ± 2.52	0.637
Hb (g/dl)	13.28 ± 4.24	12.41 ± 3.08	0.478
Neutrophil (10^9^ L)	4.65 ± 2.51	4.19 ± 2.02	0.513
Lymphocyte (10^9^ L)	2.24 ± 1.10	2.13 ± 0.97	0.739
NLR	2.53 ± 2.01	2.68 ± 1.51	0.752
IG (10^9^ L)	0.033 ± 0.061	0.221 ± 0.275	0.008
IG%	0.306 ± 0.177	0.935 ± 1.249	0.042
CRP (mg/L)	5.13 ± 5.04	21.11 ± 31.92	0.043

Statistics: * Crosstabs chi-squared tests, ** Independent samples *t*-test. Abbreviations: WBC: White blood cell count; Hb: Hemoglobin; NLR: Neutrophil-to-lymphocyte ratio; IG: Immature granulocyte; IG%: Immature granulocyte percentage; CRP: C-reactive protein.

**Table 2:** Comparison of laboratory data in pediatric and adult patients according to the presence of SARS-CoV-2.

	Pediatric SARS-CoV-2			Adult SARS-CoV-2		
	Negative	Positive	*p*	Negative	Positive	*p*
CRP (Mean ± SD)	4.77 ± 3.69	80.5 ± 91.21	<0.001	5.36 ± 5.80	14.13 ± 11.58	0.008
WBC (10^9^ L) (Mean ± SD)	8.96 ± 3.21	8.47 ± 6.25	0.847	7.39 ± 2.58	7.24 ± 1.92	0.849
NLR (Mean ± SD)	2.11 ± 1.72	1.88 ± 1.71	0.856	2.67 ± 2.09	2.78 ± 1.52	0.850
IG (10^9^ L) (Mean ± SD)	0.062 ± 0.113	0.23 ± 0.007	0.046	0.023 ± 0.018	0.220 ± 0.291	0.014
IG% (Mean ± SD)	0.304 ± 0.196	3.800 ± 2.687	<0.001	0.307 ± 0.172	0.598 ± 0.398	0.009

Statistics: Independent samples *t*-test. Abbreviations: WBC: White blood cell count; Hb: Hemoglobin; NLR: Neutrophil-to-lymphocyte ratio; IG: Immature granulocyte; IG%: Immature granulocyte percentage; CRP: C-reactive protein.

**Table 3:** Evaluation of correlation between CRP, IG, and IG% values.

		IG	IG%
CRP	r	0.279	0.892
	*p*	0.014	<0.001

Statistics: Pearson correlation test.

**Table 4:** Determination of cut-off points in hematological parameters for the diagnosis of SARS-CoV-2 infection in patients presenting with dyspeptic complaints.

	Cut-Off Value	AUC	Sensitivity	Specificity	Asymptomatic 95% Confidence Interval	*p*
IG (10^9^ L)	≥0.045	0.751	0.684	0.854	0.601–0.901	0.001
IG%	≥0.650	0.740	0.526	0.955	0.586–0.893	0.001
CRP (mg/L)	≥12.4	0.679	0.632	0.914	0.499–0.859	0.020

Statistics: ROC curve analysis was performed to determine the best cutoff points for GI, IG%, and CRP values to predict SARS-CoV-2 infection. Abbreviations: IG: Immature granulocyte; IG%: Immature granulocyte percentage; CRP: C-reactive protein; AUC: area under the curve.

**Table 5:** Risk analysis for COVID-19 diagnosis with logistic regression analysis in patients presenting with dyspeptic complaints.

	OR	95% CI	*p*	Risk
IG (≥0.045 × 10^9^ L)	8.546	2.854–25.596	<0.001	Present
IG% (≥0.650)	23.611	6.135–90.877	<0.001	Present
CRP (≥12.4 mg/L)	18.171	4.915–67.181	<0.001	Present

Statistics: Whether GI, G%, and CRP values pose a risk for SARS-CoV-2 infection was evaluated by logistic regression analysis. Abbreviations: IG: Immature granulocyte; IG%: Immature granulocyte percentage; CRP: C-reactive protein; OR: Odds ratio.

## Data Availability

The datasets used and/or analysed during the current study are available from the corresponding author on reasonable request.
